# Asthma increases the risk of herpes zoster: a nested case–control study using a national sample cohort

**DOI:** 10.1186/s13223-020-00453-x

**Published:** 2020-06-23

**Authors:** So Young Kim, Dong Jun Oh, Hyo Geun Choi

**Affiliations:** 1 Department of Otorhinolaryngology-Head & Neck Surgery, CHA Bundang Medical Center, CHA University, Seongnam, South Korea; 2 Department of Internal Medicine, Asan Medical Center, University of Ulsan College of Medicine, Seoul, South Korea; 3 Department of Otorhinolaryngology-Head & Neck Surgery, Hallym University College of Medicine, Sacred Heart Hospital, 22, Gwanpyeong-ro 170beon-gil, Dongan-gu, Anyang-Si, Gyeonggi-do 14068 Republic of Korea; 4 Hallym Data Science Laboratory, Hallym University College of Medicine, Anyang, Republic of Korea

**Keywords:** Asthma, Herpes Zoster, Risk factors, Case–control studies, Cohort studies

## Abstract

**Background:**

This study aimed to complement previous studies on the risk of herpes zoster in the asthmatic adult population.

**Methods:**

The Korean Health Insurance Review and Assessment Service—National Sample Cohort (HIRA-NSC) from 2002 through 2013 was used. A total of 64,152 participants with herpes zoster were matched for age, sex, income, region of residence, hypertension, diabetes, and dyslipidemia with 239,780 participants who were included as a control group. In both the herpes zoster and control groups, previous history of asthma were investigated. The crude and adjusted odds ratios (ORs) and 95% confidence intervals (CI) of asthma for herpes zoster were analyzed using unconditional logistic regression analysis. Subgroup analyses were conducted according to age and sex.

**Results:**

Approximately 16.2% (9728/59,945) and 12.8% (30,752/239,780) of participants in the herpes zoster and control groups, respectively, had a previous history of asthma (P < 0.001). The herpes zoster group demonstrated a 1.32-times higher odds of asthma than the control group (95% CI 1.28–1.35, P < 0.001). The increased odds of asthma in the herpes zoster group persisted in all the age and sex subgroups.

**Conclusions:**

The odds for asthma were higher in the herpes zoster group.

## Background

Herpes zoster is defined as an infectious diseases caused by varicella zoster virus reactivation [1]. Approximately 30% of the general population has herpes zoster infection for their lifetime. The incidence of herpes zoster increases with advanced age [2]. The annual incidence rate of herpes zoster is 4.47 per 1000 person-years in the US population across all ages (95% confidence interval [95% CI] 4.44–4.50) [3]. In Korea, the prevalence of herpes zoster is 10.4 per 1000 person-years and 18.54 per 1000 person-years in the population of all ages and in the population ≥ 50 years old, respectively [4]. Although the incidence of herpes zoster is high and has been increasing, prevention of herpes zoster infection is not sufficiently effective. The effectiveness of the herpes zoster vaccine was estimated to be approximately 0.48 in the US population ≥ 65 years old (95% CI 0.39–0.56) [5]. The seroprevalence of varicella antibodies is as high as 97.8% in the US population (95% CI 97.1–98.3%) [6]. However, only a small number of individuals in these varicella-seropositive populations develop herpes zoster. Therefore, there might be triggering factors that reactivate latent herpes zoster infections. Several factors that make individuals susceptible to herpes zoster have been proposed including diabetes, depression, and asthma [7].

Asthma is a lower airway disease involving reversible airflow obstruction accompanied by airway hyperresponsiveness and remodeling. The global prevalence of asthma has been reported to be approximately 4.3–4.5% [8]. In Korea, approximately 2.0% of adult population was diagnosed as asthma [9]. The pathophysiologic mechanisms of asthma are largely believed to be attributed to an abnormally excessive immune response of T helper type 2 (Th2) cells. In addition, innate immune problems, including deficient induction of interferon-λ (IFN-λ), have been described [10]. These multiple innate immune defects have been found to be related to antiviral signaling pathways [11]. From these findings, several studies have reported an increased susceptibility to viral infections in asthma patients [12]. A relationship between asthma and herpes zoster has been proposed in several studies [13–17].

However, few previous studies matched case and control groups by socioeconomic factors and past medical history. Because asthma patients have demonstrated a higher incidence of past medical history than those in control groups, these potential confounders need to be matched for between asthma and control groups in studies of adult populations [17]. In addition, no prior study has reported an association between asthma and herpes zoster infection in a Korean population. Because the prevalence of herpes zoster and vaccination rates are variable among ethnicities, the association between asthma and herpes zoster may be different among various ethnicities [18]. This study hypothesized that asthma might increase the risk of herpes zoster infection in an adult population independent of socioeconomic status and past medical history. To prove this, a large, nationwide, representative cohort was analyzed to determine the odds for asthma in herpes zoster patients.

## Materials and methods

### Study population and data collection

The Ethics Committee of Hallym University (2017-I102) approved the use of these data. Written informed consent was waived by the institutional review board.

This national cohort study relies on data from the Korean Health Insurance Review and Assessment Service—National Sample Cohort (HIRA-NSC). The Korean National Health Insurance Service (NHIS) selects samples directly from the entire population database to prevent nonsampling errors. The details of the methods used to perform these procedures are provided by the National Health Insurance Sharing Service [19].

### Participants selection

Out of 1,125,691 cases with 114,369,638 medical claim codes, we included participants who were diagnosed with herpes zoster (ICD-10: B02). Among them, we only included participants who were treated ≥ 2 times or who were treated with antiviral medication ≥ 1 time. From 2002 through 2013, 64,152 participants with herpes zoster were selected.

Asthma was defined by the ICD-10 codes for asthma (ICD-10: J45) and status asthmaticus (J46). We selected participants who were treated for asthma ≥ 2 times and who were treated with corticosteroids, a steroid inhaler, long-acting muscarinic antagonists (LAMA), leukotriene receptor antagonists (LTRA), and xanthine (n = 230,764). This method has been modified from a previous study [20].

**Fig. 1:**
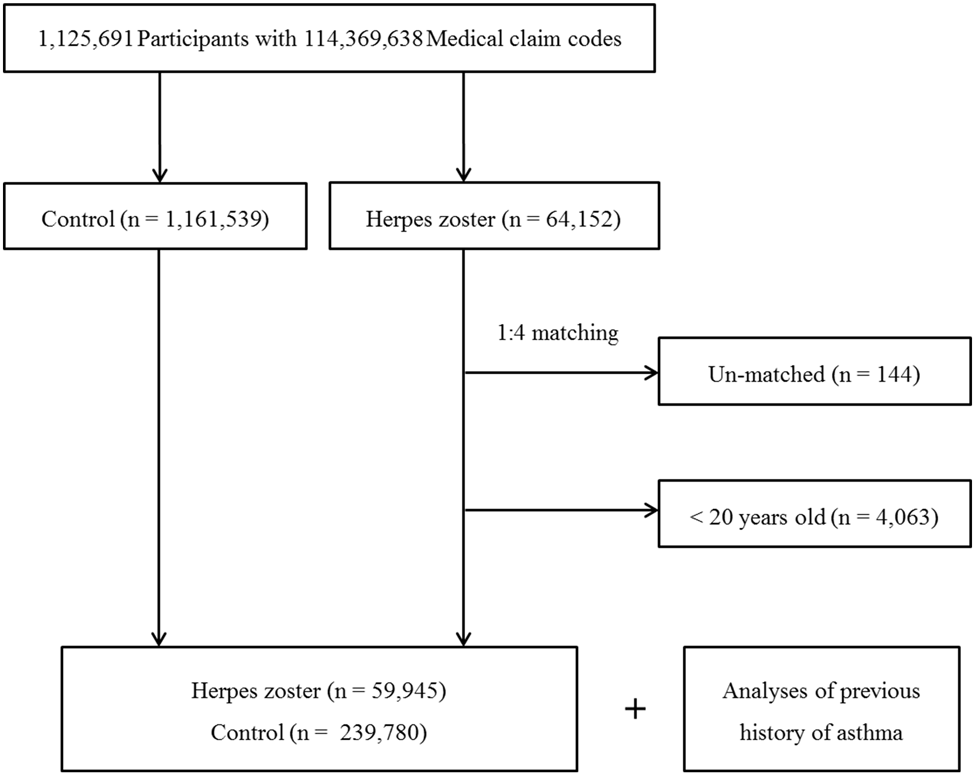
A schematic illustration of the participant selection process that was used in the present study. Out of a total of 1,125,691 participants, 59,945 of asthma participants were matched with 239,780 control participants for age, group, sex, income group, region of residence, and the past medical history

The herpes zoster participants were matched 1:4 with participants (control group) who were never diagnosed with herpes zoster from 2002 through 2013 among this cohort. The control group was selected from the mother population (n = 1,161,539). The characteristics of the matched participants were identified including age, group, sex, income group, region of residence, and past medical history (hypertension, diabetes, and dyslipidemia) [21]. To prevent selection bias when selecting the matched participants, the control group participants were sorted using a random number order, and they were then selected from top to bottom. It was assumed that the matched control participants were involved at the same time as each of the matched asthma participants (index date). Therefore, participants in the control group who died before the index date were excluded. The herpes zoster participants for whom we could not identify enough matching participants were excluded (n = 144). We excluded participants who were younger than 20 years old (n = 4063). Finally, 1:4 matching resulted in the inclusion of 59,945 of herpes zoster participants and 239,780 control participants (Fig. 1). However, they were not matched for ischemic heart disease, cerebral stroke, or depression history because strict matching increased the participant drop out rate due to a lack of control participants. After matching, we analyzed the previous history of asthma in both the herpes zoster and control groups.

### Variables

The age groups were classified using 5-year intervals: 20–24, 25–29, 30–34…, and 85+ years old. A total of 14 age groups were designated. The income groups were initially divided into 41 classes (one health aid class, 20 self-employment health insurance classes, and 20 employment health insurance classes). These groups were recategorized into 11 classes (class 1 [lowest income]-11 [highest income]). Participants’ region of residence was divided into 16 areas according to administrative districts. These regions were regrouped into urban (Seoul, Busan, Daegu, Incheon, Gwangju, Daejeon, and Ulsan) and rural (Gyeonggi, Gangwon, Chungcheongbuk, Chungcheongnam, Jeollabuk, Jeollanam, Gyeongsangbuk, Gyeongsangnam, and Jeju) areas.

The participants’ past medical history was evaluated using ICD-10 codes. For the accuracy of diagnosis, participants were considered to have hypertension (I10 and I15), diabetes (E10-E14), and dyslipidemia (E78) if they were treated ≥ 2 times. They were considered to have ischemic heart disease (I24 and I25) and cerebral stroke (I60–I66) if they were treated ≥ 1 time. Depression was defined using the ICD-10 codes F31 (bipolar affective disorder) through F39 (unspecified mood disorder) if these codes were indicated by a psychiatrist ≥ 2 times.

### Statistical analyses

To analyze the odds ratio (OR) of asthma for herpes zoster, unconditional logistic regression analysis was used. In this analysis, crude (simple) and adjusted (age, sex, income, region of residence, hypertension, diabetes, dyslipidemia, ischemic heart disease, cerebral stroke, and depression histories) models were used, and 95% CIs was calculated.

For the subgroup analyses, we divided the participants by age (< 40 years old, ≥ 40 years old & < 60 years old, and ≥ 60 years) and sex (male and female).

Two-tailed analyses were conducted, and P values less than 0.05 were considered to indicate significance. The results were statistically analyzed using SPSS v. 22.0 (IBM, Armonk, NY, USA).

## Results

The rate of asthma was higher in the herpes zoster group (16.2% [9728/59,945]) than in the control group (12.8% [30,752/239,780], P < 0.001, Table 1). The general characteristics (age, sex, income, region of residence, and history of hypertension, diabetes, or dyslipidemia) of the participants in both groups were exactly same due to matching (P = 1.000). The rates of ischemic heart disease, cerebral stroke, and depression were higher in the herpes zoster group (each P < 0.05).

**Table 1:** General characteristics of participants

Characteristics	Total participants		
	Herpes zoster (n, %)	Control group (n, %)	*P* value
Age (years old)			1.000
20–24	1829 (3.1)	7316 (3.1)	
25–29	2801 (4.7)	11,204 (4.7)	
30–34	3422 (5.7)	13,688 (5.7)	
35–39	3899 (6.5)	15,596 (6.5)	
40–44	4637 (7.7)	18,548 (7.7)	
45–49	6203 (10.3)	24,812 (10.3)	
50–54	7991 (13.3)	31,964 (13.3)	
55–59	7272 (12.1)	29,088 (12.1)	
60–64	6311 (10.5)	25,244 (10.5)	
65–69	5836 (9.7)	23,344 (9.7)	
70–74	4685 (7.8)	18,740 (7.8)	
75–79	2870 (4.8)	11,480 (4.8)	
80–84	1424 (2.4)	5696 (2.4)	
85+	765 (1.3)	3060 (1.3)	
Sex			1.000
Male	23,865 (39.8)	95,460 (39.8)	
Female	36,080 (60.2)	144,320 (60.2)	
Income			1.000
1 (lowest)	9152 (15.3)	36,608 (15.3)	
2	8383 (14.0)	33,532 (14.0)	
3	9910 (16.5)	39,640 (16.5)	
4	13,149 (21.9)	52,596 (21.9)	
5 (highest)	19,351 (32.3)	77,404 (32.3)	
Region of residence			1.000
Urban	28,575 (47.7)	114,300 (47.7)	
Rural	31,370 (52.3)	125,480 (52.3)	
Hypertension			1.000
Yes	23,708 (39.5)	94,832 (39.5)	
No	36,237 (60.5)	144,948 (60.5)	
Diabetes			1.000
Yes	12,537 (20.9)	50,148 (20.9)	
No	47,408 (79.1)	189,632 (79.1)	
Dyslipidemia			1.000
Yes	18,386 (30.7)	73,544 (30.7)	
No	41,559 (69.3)	166,236 (69.3)	
Ischemic heart disease			<0.001*
Yes	4523 (7.5)	15,488 (6.5)	
No	55,422 (92.5)	224,292 (93.5)	
Cerebral stroke			0.003*
Yes	6782 (11.3)	26,100 (10.9)	
No	53,163 (88.7)	213,680 (89.1)	
Depression			<0.001*
Yes	6703 (11.2)	22,517 (9.4)	
No	53,242 (88.8)	217,263 (90.6)	
Asthma			<0.001*
Yes	9728 (16.2)	30,752 (12.8)	
No	50,217 (83.8)	209,028 (87.2)	

*Chi square test. Significance at P < 0.05

The herpes zoster participants have the 1.32 times higher chance of previous asthma histories in adjusted model (95% CI 1.28–1.35, P < 0.001, Table 2).

**Table 2:** Crude and adjusted odd ratios (95% confidence interval) of asthma for herpes zoster

Characteristics	Asthma			
	Crude	P-value	Adjusted^†^	P-value
Herpes zoster	1.32 (1.29–1.35)	< 0.001*	1.32 (1.28–1.35)	< 0.001*
Control	1.00		1.00	

* Logistic regression analyses, Significance at P < 0.05

^†^ Adjusted model for age, sex, income, region of residence, hypertension, diabetes, dyslipidemia, ischemic heart disease, cerebral stroke, and depression histories

In each subgroup according to age and sex, the herpes zoster participants have the higher OR of previous asthma histories in crude and adjusted model (each P < 0.05, Table 3). The adjusted OR was 1.22 (95% CI 1.07–1.40) in men < 40 years old, 1.40 (95% CI 1.28–1.53) in women < 40 years old, 1.47 (95% CI 1.36–1.59) in men who were 40–59 years old, 1.32 (95% CI 1.26–1.38) in women who were 40–59 years old, 1.33 (95% CI 1.25–1.41) in men ≥ 60 years old, and 1.26 (95% CI 1.20–1.32) in women ≥ 60 years old.

**Table 3:** Subgroup analysis of crude and adjusted odd ratios (95% confidence interval) of asthma for herpes zoster according to age and sex

Characteristics	Asthma			
	Crude	P-value	Adjusted^†^	P-value
Age < 40 years old, men (n = 26,685)				
Herpes zoster	1.23 (1.07–1.41)	0.003*	1.22 (1.07–1.40)	0.004*
Control	1.00		1.00	
Age < 40 years old, women (n = 33,070)				
Herpes zoster	1.41 (1.29–1.54)	< 0.001*	1.40 (1.28–1.53)	< 0.001*
Control	1.00		1.00	
Age ≥ 40 years old & < 60 years old, men (n = 49,630)				
Herpes zoster	1.48 (1.37–1.60)	< 0.001*	1.47 (1.36–1.59)	< 0.001*
Control	1.00		1.00	
Age ≥ 40 years old & < 60 years old, women (n = 80,885)				
Herpes zoster	1.32 (1.26–1.39)	< 0.001*	1.32 (1.26–1.38)	< 0.001*
Control	1.00		1.00	
Age ≥ 60 years old, men (n = 43,010)				
Herpes zoster	1.34 (1.26–1.42)	< 0.001*	1.33 (1.25–1.41)	< 0.001*
Control	1.00		1.00	
Age ≥ 60 years old, women (n = 66,445)				
Herpes zoster	1.27 (1.21–1.33)	< 0.001*	1.26 (1.20–1.32)	< 0.001*
Control	1.00		1.00	

* Logistic regression analyses, Significance at P < 0.05

^†^Adjusted model for age, sex, income, region of residence, hypertension, diabetes, dyslipidemia, ischemic heart disease, cerebral stroke, and depression histories

## Discussion

Patients with herpes zoster showed a higher odds of asthma than those in the control group that was independent of age, sex, income, region of residence, and past medical history of diabetes, hypertension, dyslipidemia, ischemic heart disease, and stroke. Few previous studies have used control groups matched by socioeconomic status and past medical history. The odds of asthma in individuals with herpes zoster were evaluated according to age and sex. In addition, this is the first study to evaluate the association between asthma and herpes zoster in a Korean population.

Several previous studies have demonstrated an association between asthma and herpes zoster [13–17]. Three case–control studies have reported that children with asthma have an increased risk of herpes zoster infection compared with those in control groups [13–15]. Two additional case–control studies have demonstrated the risk of herpes zoster in asthmatic adults [16, 17]. The risk of herpes zoster was 1.48 times higher in an asthmatic adult population than in an age- and sex-matched control group (95% CI = 1.36–1.62) [17]. Another case–control study reported that the risk of herpes zoster was 1.70 times higher in asthmatic adults ≥ 50 years old than in individuals in an age- and sex-matched control group (95% CI 1.20–2.42, P = 0.003) [16]. These results were comparable to the findings of the present study. The slightly lower odds of 1.32 in this study than that in previous studies might be explained because the control group was stringently matched for socioeconomic status and past medical history. A number of plausible pathophysiologic mechanisms including immunologic causes may increase the risk of herpes zoster in asthmatic adults.

Impaired immune responses in asthma patients could increase their susceptibility to herpes zoster infection. Asthma is associated with skewed Th1/Th2 immunity, which leads to Th2 predominant conditions. On the other hand, reciprocal Th1 immune insufficiency could occur in asthma patients. The varicella zoster virus infection is predominantly related to T cell-mediated cellular immunity [22]. The impaired cell-mediated immunity associated with herpes zoster infection is not confined to local skin. The fluid in skin blisters and peripheral blood of herpes zoster patients has low levels of the Th1 cytokines interleukin (IL)-2 and tumor necrosis factor-α and high levels of the Th2 cytokines IL-10 and IL-4 compared with the levels of these cytokines in samples from a control group [23]. Thus, Th1 immune deficiency in asthma patients could increase their susceptibility to herpes zoster infection. In addition, impaired innate immunity in asthma patients could increase the risk of herpes zoster infection. Asthma patients have impaired innate immune responses and mucosal defense systems, which result in an altered lower respiratory microbiome [24]. Likewise, herpes zoster infection is also associated with deficiencies in the antiviral properties of the innate immune system. Multiple innate immune system-related cytokines including IFN-α of plasmacytoid dendritic cells contribute to the defense mechanisms against varicella zoster infection [25]. On the other hands, the acquisition of immune system by the prior primary viral infection or vaccination, such as for varicella zoster virus, had been suggested to have protective effects on asthma in aspects of delayed onset [26] and clinical outcomes with milder symptoms and alleviation of atopy [27, 28].

Asthma can indirectly increase the risk of herpes zoster infection via unidentified comorbidities. Asthma is associated with numerous comorbid conditions including chronic obstructive pulmonary disease, diabetes, and depression [29–31]. These comorbidities have also been suggested to be risk factors for herpes zoster [7]. Metabolic and inflammatory pathways have been suggested to be involved in the development of asthma and herpes zoster infection [14, 16]. These common pathophysiologic pathways could link asthma and herpes zoster infection.

The increased risk of herpes zoster infection in asthmatic patients was consistent in all age and sex subgroups in the present study. Although the risk of herpes zoster infection is high in the elderly population, an association between asthma and herpes zoster infection was present in all the adult age groups. Similar to the present results, a previous study also demonstrated higher hazard ratios of herpes zoster infection in an asthmatic adult population > 20 years old [17]. Because the risk of herpes zoster infection was elevated in young adult as well as elderly with asthma, the vaccination of herpes zoster infection might be beneficial in wide range of age groups from young adult to elderly asthmatic patients.

One of the advantages of this study compared to the abovementioned previous studies is that the control group was matched for socioeconomic status and past medical history. In addition, this study used a representative, nationwide population and objective inclusion criteria for both asthma and herpes zoster. The NHIS data includes all the medical records of the Korean population without exception. The unified national health care system using the NHIS allows tracing of the medical records of all Koreans including medical claim codes and prescriptions. Based on this mother population, a specialized statistician extracted a representative sample cohort including data regarding age, sex, income, and region of residence. Cohort studies using medical claim codes have merit because of the relatively objective disease classification compared to questionnaire-based survey studies. However, comparable medical accessibility and availability between the study and control groups must be attained to minimize selection bias because the NHIS data collects medical records that are dependent on health care visits. Thus, matching and adjustment for socioeconomic status are important in analysis of NHIS day.

The objective inclusion criteria of ICD-10 codes and medication history improved the fidelity of the current study. However, the severity and management of both asthma and herpes zoster infection could not be determined using the NHIS data. The heterogeneity in the severity or type of disease leaves the exact pathophysiologic link between asthma and herpes zoster to be further elucidated. For instance, asthma has various endotypes with respectively different pathophysiological mechanisms. However, a previous study demonstrated that the risk of herpes zoster infection in asthma patients persisted independent of the definition of asthma that was used [15]. In addition, the status of asthma control could not be determined in this study. A prior study described that uncontrolled asthma patients with recurrent emergency department visits or admissions had a higher risk of herpes zoster infection than well-controlled asthma patients [13]. Moreover, asthma patients treated with regular inhaled corticosteroids had a higher risk of herpes zoster infection than those who did not use this treatment for regular control [13]. This study could not differentiate the steroid medications used in asthma patients; however, the prescription of steroids can influence to the risk of herpes zoster infection in asthma patients. Similar limitations regarding the heterogeneity of the severity and type of disease are also present in the evaluation of herpes zoster infection. Severe herpes zoster patients or immunocompromised patients were reported to have more frequent outpatient visits and hospitalizations [4]. Lastly, possible confounders including obesity, smoking history, and alcohol consumption that were not considered might restrict interpretation of the present study.

## Conclusion

Asthma increased the risk of herpes zoster infection in an adult population compared to the risk of herpes zoster infection in a control group matched for socioeconomic status and past medical history. This increased risk was consistent according to age and sex.

## Data Availability

The datasets supporting the conclusions of this article are available in the database of National health Insurance Sharing Service (NHISS)https://nhiss.nhis.or.kr/. NHISS allows all of this data for the any researcher who promises to follow the research ethics with some cost. If you want to access the data of this article, you could download it from the website after promising to follow the research ethics.
